# Effect of conditioned medium of umbilical cord-derived mesenchymal stem cells as a culture medium for human granulosa cells: An experimental study

**DOI:** 10.18502/ijrm.v19i12.10054

**Published:** 2022-01-12

**Authors:** Kanadi Sumapraja, Andon Hestiantoro, Isabella Kurnia Liem, Arief Boediono, Teuku Z Jacoeb

**Affiliations:** ^1^Division of Reproductive Immunoendocrinology, Department of Obstetrics and Gynecology, Faculty of Medicine Universitas Indonesia, Dr. Cipto Mangunkusumo Hospital, Jakarta, Indonesia.; ^2^Cluster of Human Reproduction, Fertility and Family Planning, the Indonesian Medical Education and Research Institute Universitas Indonesia, Jakarta, Indonesia.; ^3^Department of Anatomy, Faculty of Medicine Universitas Indonesia, Jakarta, Indonesia.; ^4^Stem Cell Medical Technology Integrated Service Unit, Dr. Cipto Mangunkusumo Hospital, Jakarta, Indonesia.; ^5^Faculty of Veterinary Medicine, Bogor Agricultural University, Bogor, Indonesia.

**Keywords:** Conditioned medium, BAX, Survivin, GDF9, IGF-1.

## Abstract

**Background:**

The umbilical cord-derived mesenchymal stem cells conditioned medium (UC-MSCs-CM) produces secretomes with anti-apoptotic properties, and has the potential to prevent apoptosis of granulosa cells (GC) during controlled ovarian hyperstimulation.

**Objective:**

To observe the effect of UC-MSCs-CM on the interaction between pro- and anti-apoptotic proteins and the influence of growth differentiation factor 9 (GDF9) production in GC.

**Materials and Methods:**

UC-MSCs-CM was collected from umbilical cord stem cell culture on passage 4. GC from 23 women who underwent in vitro fertilization were cultured and exposed to UC-MSCs-CM for 24 hr. Then RNA of the GC was extracted and the mRNA expression of BCL-2 associated X (BAX), survivin and GDF9 were analysed using quantitative real-time PCR. The spent culture media of the GC were collected for measurement of insulin growth factor 1 using ELISA.

**Results:**

The expression of BAX was significantly different after UC-MSCs-CM exposure (4.09E-7 vs. 3.74E-7, p = 0.02). No significant changes occurred in survivin, BAX/survivin ratio, and GDF9 expression after UC-MSCs-CM exposure (p > 0.05). The IGF-1 level of the CM was significantly higher after the CM was used as a culture medium for GC (2.28 vs. 3.07 ± 1.72, p ≤ 0.001). A significant positive correlation was found between survivin and GDF9 (r = 0.966, p ≤ 0.001).

**Conclusion:**

IGF-1 produced by UC-MSCs-CM can work in paracrine fashion through the IGF receptor, which can inhibit BAX and maintain GDF9 production. Moreover, under the influence of UC-MSCs-CM, GC are also capable of producing IGF-1, which can impact GC through autocrine processes.

## 1. Introduction 

The cumulus granulosa cells (GC) are the most important cells for the survival of the oocyte. There is strong two-way communication between cumulus GC and oocytes that determines oocyte survival (1-3). Cumulus GC have an essential role in oocyte maturation by secreting growth factors (insulin-like growth factor, IGF; growth hormone releasing factor, GHRF; and epidermal growth factor, EGF) and oocyte secreted factors (growth differentiation factor 9, GDF9; and bone morphogenetic protein 15, BMP15). The level of expression of GDF9 and BMP15 is associated with oocyte maturation rate, fertility, embryo quality and pregnancy outcome (2, 4, 5).

Controlled ovarian hyperstimulation is an essential step in the in vitro fertilization (IVF) procedure. It can increase the number of oocytes collected in one cycle of stimulation. However, the use of particular regimens in controlled ovarian hyperstimulation, such as GnRH antagonist, can induce GC apoptosis (6). The apoptosis of GC can impair oocyte maturation and thereby impact the number of collected oocytes, fertilization rate, embryo development and pregnancy rate (7, 8).

The use of stem cells as therapeutic agents in regenerative medicine has become popular. Stem cells have the potential to regenerate damaged tissue through their capability of self-renewal and differentiation to other cells (9, 10). Mesenchymal stem cells (MSCs) have been extensively used to treat dysfunction of various organs with varied results. It is believed that MSCs might be able to repair damaged tissue by directly replacing the damaged cells or through paracrine effects from their products (secretomes and exosomes). The medium of MSC culture containing secretomes and exosomes is called the conditioned medium (CM) (11, 12). IGF-1 is one of the secretomes produced by MSCs, which shows anti-apoptotic properties (13). Previous studies have shown the capability of CM to help restore ovulatory functions after the ovary has been artificially shut down by chemotherapy (14, 15). Recently, the use of stem cells has shifted from cell-based therapy to non-cell based therapy in order to prevent the potential disadvantages of direct stem cell treatment (13).

This research investigated the effect of umbilical cord-derived mesenchymal stem cell conditioned medium (UC-MSCs-CM) containing IGF-1 on GC from normal responders, to determine the interactions between apoptosis components represented by BCL-2 associated X protein (BAX), survivin and the BAX/survivin ratio, and GC function represented by GDF9.

## 2. Materials and Methods

### Study setting

This experimental study was conducted to evaluate the apoptosis effect of UC-MSCs-CM on human GC from normal responder women undergoing IVF. The study took place at Yasmin Clinic, Dr. Cipto Mangunkusumo General Hospital, Indonesia, between February 2017 and March 2018.

### Subjects

IVF women who underwent ovarian stimulation and were normal responders were eligible to be subjects. Informed consent was obtained from all participants. Women with incomplete baseline data or failed ovum pick-up were excluded. Twenty-three women were recruited according to the following inclusion criteria: (1) aged < 40 yr; (2) minimum three oocytes; (3) anti-mullerian hormone > 1.4 mIU/ml and antral follicle count < 10; (4) responsive with 225 units of recombinant follicle-stimulating hormone (FSH); (5) GC of at least 1,000,000/ml; (6) no reproductive disorder.

### Data collection

Along with carrying out the ovum pick-up procedure, the doctor extracted the follicular fluid containing oocytes and GC from the women under anesthesia. The follicular fluid was then processed by an embryologist. The oocytes were processed in the next stage of IVF and the GC were cultured.

### GC culture

The GC were counted using a hemocytometer. The GC were then divided into two parts. The first represented the baseline (D0) and the second group represented post-intervention with CM (D1). 500,000 cells/ml GC were cultured using alpha minimal essential medium ( α MEM) (Gibco ${}^{\text{TM}}$ , USA), 10% platelet rich plasma and 1% penicillin/streptomycin (Gibco ${}^{\text{TM}}$ , USA) in a 5% CO ${}_{2}$ incubator at 37°C for 24 hr. On day one of the culture, the GC media were changed using UC-MSCs-CM and incubated in a 5% CO ${}_{2}$ incubator at 37°C for 24 hr. After 24 hr, the GC were harvested using 0.25% Tripsin-EDTA (Gibco ${}^{\text{TM}}$ , USA), and then the RNA was isolated. The media were collected for IGF-1 measurement.

UC-MSCs-CM was collected from umbilical cords. Stem cells were cultured using αMEM (Gibco ${}^{\text{TM}}$ , USA), 10% platelet rich plasma and 1% penicillin/streptomycin (Gibco ${}^{\text{TM}}$ , USA) in a 5% CO ${}_{2}$ incubator at 37°C until passage 4. The spent culture media were then collected and used for GC culture.

RNA extraction and cDNA synthesis were carried out through quantitative real-time polymerase chain reaction (RT-PCR) with GC culture. The RNA from the baseline and post-intervention groups was isolated using QIAamp RNA Blood Mini Kit according to the protocol in QIAamp RNA Blood Mini Handbook (Qiagen, Germany). The RNA concentration level was measured by Nano Drop (Thermo Fisher Scientific, USA). Subsequently, cDNA synthesis was performed using Quantitect reverse transcription (Qiagen, USA). Quantitect SYBR Green RT-PCR was used with the following profile: initial activation (one cycle at 95°C for 15 min), denaturation (40 cycles at 94°C for 15 sec), annealing (40 cycles at TM primer temperature for 30 sec), and extension (40 cycles at 72°C for 30 sec). All of the procedures were carried out according to the manufacturer's instructions. 20 ng/μl of cDNA was used. A standard curve for quantification of the mRNA expression for each subject was calculated using G block. The RT-PCR was performed using Prime Pro 48 Real-Time PCR Machine (Cole-Parmer, UK). Primer sequences can be seen in table I.

The spent media from the GC culture with UC-MSCs-CM and UC-MSCs-CM media stock were collected. The IGF-1 spent media were measured using Human IGF-1 Immunuassay ELISA (Quantikine, USA). The IGF-1 level of the CM was measured with the ELISA sandwich method using monoclonal antibodies specific to IGF-1 (mouse monoclonal IgG1), which were coated to the base of the well. Secondary antibodies conjugated with biotin were added, and finally, the enzyme (horseradish peroxidase) which conjugated with streptavidin, was added in order to create a reaction between the enzyme and substrate. The concentration of IGF-1 was measured with an ELISA reader at the wavelength of 450 nanometer.

**Table 1:** Primer sequence for qPCR


**Gene**	**Accession No.**	**Primer sequence (5'-3')**	**Size (bp)**	**Temperature (°C)**
*GDF9 *	NM_0011543308.3	Forward: GAG TGT GAG CTC CAT GAC TTT Reverse: CCC TTT ACA GTA TCG AGG GTT G	107	62
*BAX*	NM_001291428.2	Forward: CGG CCT CCT CTC CTA CTT Reverse: GCC TCA GCC CAT CTT CTT C	106	56
*Survivin*	NM_001168.3	Forward: GGA TCA CGA GAG AGG AAC ATA AA Reverse: GGC TCT TTC TCT GTC CAG TTT	104	54
*GDF9*: Growth differentiation factor 9, *BAX*: BCL-2 associated X				

### Ethical considerations

This study was approved by The Health Research Ethics Committee, Universitas Indonesia and Dr. Cipto Mangunkusumo General Hospital (Code: 197/UN2.F1/ETIK/2017). Written informed consent was obtained from all participants.

### Statistical analysis

Data were statistically analyzed using the Statistical Package for the Social Sciences (SPSS) version 22.0. The primary data of the women such as age, body mass index, dose of FSH and number of oocytes were analyzed. The correlation ratio of BAX, survivin, and IGF-1 were compared with GDF9 expression using the Mann-Whitney test.

## 3. Results

**Figure 1:**
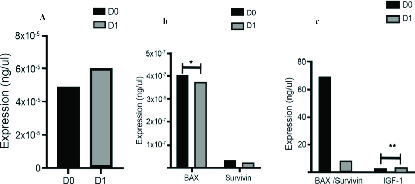
The changes in mRNA expression before and after UC-MSCs-CM exposure and the level of IGF-1 in UC-MSCs-CM. (a) The change in mRNA expression in GDF9 showing no statistical difference on day 0 vs. day 1; (b) The mRNA expression in survivin and BAX with a significant reduction in BAX expression after exposure to UC-MSCs-CM (p < 0.05); (c) The mRNA expression ratio of BAX/survivin and the level of IGF-1 showing a significant increase in IGF-1 levels of UC-MSCs-CM after exposure to GC.

GC from a total of 23 women were defined into two groups: baseline and exposed to UC-MSCs-CM. We evaluated the mRNA expression in the GC (Figure 1). In this study we found a statistically significant reduction of BAX expression in GC after exposure to UC-MSCs-CM (4.09E-7 vs. 3.74E-7, p = 0.02). The IGF-1 level in the CM was significantly higher after being used for GC culture (2.28 vs. 3.07 ± 1.72, p ≤ 0.001).

To compare the contributions of all variables, we counted the difference between day 1 and day 0 before UC-MSCs-CM exposure. The differences in the expression of BAX and survivin, and the level of IGF-1 compared to GDF9 expression, are shown in table II. The data showed a strong positive correlation between survivin and GDF9 (r = 0.996, p ≤ 0.001), while the other variables did not show any significant correlations.

**Table 2:** The correlation between Δ BAX and Δ survivin, and between Δ IGF-1 and Δ GDF9 (n = 20)


	**Δ GDF9**	
**Δ BAX**	r = 0.177	p = 0.45
**Δ Survivin**	r = 0.996*	p < 0.001**
**Δ IGF-1**	r = 0.084	p = 0.72
*Positive and strong correlation, **Statistically significant, BAX: BCL-2 associated X protein, GDF: Growth differentiation factor, IGF-1: Insulin-like growth factor-1, r: Correlation coefficient, Δ: Differentiation of gene expression before and after UC-MSCs-CM exposure		

## 4. Discussion

This study showed a statistically significant BAX reduction in GC after being exposed to CM for 24 hr (p = 0.02). BAX is an important pro-apoptotic protein of the BCL-2 family which can be activated either by intrinsic or extrinsic pathways (15). It seems that CM has the potential to inhibit apoptosis by blocking BAX activation. However, this study failed to show significant changes in survivin, the BAX/survivin ratio, or GDF9 after exposure to CM.

It was expected that IGF-1 produced by CM would convert the pro-apoptotic activity in GC into anti-apoptotic activity by reducing BAX and increasing survivin expression (16-19). GC have three kinds of apoptotic pathways. The alternative apoptotic pathway in GC involves FSH and the activation of granzymes, which does not associate with mitochondrial activity. Therefore, the alternative pathway preserves the mitochondrial function of steroidogenesis (20). This condition might explain why IGF-1 in CM did not sufficiently enhance survivin expression. IGF-1 anti-apoptotic activity probably mainly worked through suppression of BAX and the alternative pathway. The other possibility is that BAX and survivin were essential in the GC apoptotic pathway. Therefore, BAX and survivin might have their own regulatory mechanisms that maintain their appropriate action (21-25).

Based on the individual case analysis, a large BAX reduction after CM exposure was found in participants with a very low percentage of oocyte maturation (16.67%). This initial finding prompted some speculation about various GC phenotypes among normal responder subjects. It was most likely that the subjects with poor oocyte maturation also had poor GC. The IGF-1 that was produced by UC-MSCs-CM probably worked to regenerate the GC by BAX inhibition. However, since this study used GC from normal responder subjects, the benefit on BAX reduction might have been experienced more prominently by GC that were poor quality.

On the other hand, survivin expression was found to be elevated after CM exposure in subjects who had a good percentage of oocyte maturation (100%). Meanwhile, subjects with a poor percentage of oocyte maturation (16.67%) did not show survivin elevation after CM exposure. This finding could have occurred because poor quality GC did not have sufficient mechanisms to induce survivin elevation.

No changes were seen in GDF9 production after CM exposure. GDF9 stimulates the proliferation of GC from both large and small follicles in the presence or absence of IGF-1. However, GDF9 can inhibit FSH and IGF-1-induced estradiol and progesterone production from both large and small follicles. It seemed that both IGF-1 and GDF9 worked together to regulate steroidogenesis and growth of the follicles (26). GDF9 expression was expected to be elevated if GC could maintain their function by enhancing more anti-apoptotic activity. This hypothesis was supported in this study as there was a strong positive correlation between survivin and GDF9 (p ≤ 0.001). Therefore, by increasing the anti-apoptotic protein, the survival of GC and the production of GDF9 that is responsible for the growth of the follicle and steroidogenesis can be maintained (1-3).

However, based on individual case analysis, GDF9 was found highly elevated in subjects with a poor percentage of oocyte maturation (16.67%). It seemed that poor quality GC benefited from CM not only because the secretomes and/or exosomes produced by UC-MSCs-CM inhibited the pro-apoptotic protein, but also because they prevented further reduction of anti-apoptotic proteins. The inhibition of the pro-apoptotic pathway probably can restimulate GC function in terms of GDF9 production, since GDF9 is also responsible for GC proliferation.

A statistically significant increase in IGF-1 levels was identified in CM after it was used as a culture medium for GC. This finding suggests the possibility of IGF-1 production by GC induced by CM. IGF-1 was one of the growth factors found in the secretomes of MSCs with anti-apoptotic properties (12). Initially IGF-1 in the CM was expected to decline after exposure to GC. On the contrary, this study showed that IGF-1 levels in CM increased after the CM was used as a culture medium for GC. It seemed that GC were also producing IGF-1 while being incubated by CM. Therefore, GC also had the capability to produce IGF-1, which can be used for communication between GC and oocytes (1-3). The initial concentration of IGF-1 inside the CM was 2.28 ng/mL, and this was elevated to 3.07 ± 1.72 ng/mL after exposure. However, the initial (D0) and the final (D1) concentrations of IGF-1 in the CM were still below the minimum concentration required to prevent GC from apoptosis based on a previous study (27). This meant that there was no association found between IGF-1 and BAX or survivin in this study. It was hypothesized that IGF-1 could influence GC either through paracrine or autocrine mechanisms to reduce pro-apoptotic activity, mainly by inhibiting BAX to maintain GDF9 production.

Unfortunately, this study did not evaluate the final part of the apoptotic cascade to confirm the presence of apoptosis in GC, or the regulation mechanisms of each variable (BAX, survivin and GDF9), and there was no attempt to use different kinds of CM dosage to understand dose- dependent response.

## 5. Conclusion

The use of UC-MSC-CM as a culture medium for GC can prevent GC apoptosis by inhibiting BAX expression, maintaining GDF9 production through its effect on survivin, and inducing IGF-1 production by GC. Thus, IGF-1 impacts GC through paracrine and autocrine mechanisms.

##  Conflict of Interest

No potential conflict of interest was reported by the authors.
